# Supplementary material for: Soft X-ray spectro-ptychography of boron nitride nanobamboos, carbon nanotubes and permalloy nanorods
Source: J Synchrotron Radiat. 2023 May 5;30(Pt 4):746–57. doi: 10.1107/S1600577523003399 (PMC10325009; doi:10.1107/S1600577523003399)
Supplement: Supplementary file 1 [file s-30-00746-sup1.pdf]

Soft X-ray spectro-ptychography of boron nitride nanotubes, carbon nanotubes and permalloy nanorods

Jaianth Vijayakumar,[1#*] Hao Yuan,[2§] Nicolas Mille, [1] Stefan Stanescu,[1] Sufal Swaraj,[1] Vincent Favre-Nicolin,[3,4] Ebrahim Najafi,[5] Adam P. Hitchcock, [2] and Rachid Belkhou[1]

1 Synchrotron SOLEIL, L'Orme des Merisiers, Saint-Aubin, BP 48, 91192 Gif-sur-Yvette Cedex, France

2 Department of Chemistry & Chemical Biology, McMaster University, Hamilton, Canada, L8S 4M1

3 ESRF, The European Synchrotron, 71 Avenue des Martyrs, 38000 Grenoble, France

4 Université Grenoble Alpes, Grenoble, France

5 The Chemours Company, Wilmington, Delaware 19899 US

CONTENTS

SI-1 Experimental acquisition and PyNx reconstruction parameters

SI-2 Diffraction signal before and after background subtraction

SI-3 Ptychography and spectro-ptychography of carbon nanotubes

SI-4 Ptychographic absorption and phase imaging of BN nanobamboo across the B 1s $\rightarrow\pi^*$transition

SI-5 Evaluating spatial resolution

SI-6 Comparison of diffraction images from permalloy nanorods and BN nanobamboo

SI-7 BN nanobamboo measured at N-edge (401.8 eV)

Section SI-1 Experimental acquisition and PyNX reconstruction parameters

Table S1: Experimental acquisition and reconstruction parameters for data presented in each figure

Figure	Sample	Area (µm)	# DI images	Spot size (nm)	Overlap factor (%)	E_{photon} (eV)	Dwell (ms)	Overlap factor (k)	Dose (MGy)	Reconstruction pixel (nm)	Reconstruction algorithm
1.c	BN bamboo	1.5x1.5	100x100	62	84	192.5	50	30	35	-	Integrated STXM
1.(d-e) & S6.b	BN bamboo	2x2	20x20	1000	90	192.5	200	78	55	18.9	AP*400
2.a	BN bamboo	1.2x1.2	30x30	62	35.5	187-210	100	4.7	1200	21.8@	AP*300
2.c	BN bamboo	1.2x1.2	30x30	62	35.5	399-430	100	4.7	5900	9@	AP*300
3.a , 3.b	BN bamboo	2x2	20x20	1000	90	187-198	100	78	660	21.2@	AP*300
3.c, 3.d	BN bamboo	2x2	20x20	1000	90	398-430	100	78	2300	9.7@	AP*300
4.(a-b-c) & S5	Permalloy	2x2	50x50	62	50	706	50	2.3	-	18	DM*500+AP*200
5.a	BN bamboo	0.06	50	62	-	192.5	200	-	140	-	-
5.b	BN bamboo	1.0	50	1000	-	192.5	200	-	50	-	-
6.a	CNT #1	1.6x1.6	40x40	62	68	310	100	6.8	350	15.7	AP*800
6.b	CNT #1	1.6x1.6	40x40	500	92	310	100	124	130	12.7	AP*800
6.c	CNT #1	1.6x1.6	40x40	1000	96	310	100	518	35	12.7	AP*800
6.d	CNT #1	1.6x1.6	40x40	500	68	285.4	100	6.8	14	22.1	AP*1000
6.e	CNT #1	1.6x1.6	20x20	500	84	285.4	100	30	44	22.1	AP*1000
6.f	CNT #1	1.6x1.6	10x10	500	92	285.4	100	124	130	24.3	AP*1000
S2 (a-c)	CNT #1	1.6x1.6	10x10	500	92	285.2	100	124	210	24.3	AP*1000
S2 (d-e-f)	CNT#2	1.8x1.8	30x30	1000	94	285.2	200	217	65	13.5	AP*300
S3	CNT#2	1.8x1.8	30x30	1000	94	280 - 305	200	217	8700	13.5@	AP*300
S4.(a)	BN bamboo	2x2	20x20	1000	90	187-198	100	78	1200	21.2@	AP*300
S4.(b)	BN bamboo	2x2	20x20	1000	90	398-430	100	78	5900	9.7@	AP*300
S8.(a,b)	BN bamboo	2x2	20x20	1000	90	401.8	200	78	55	9.7	AP*400

Spot size – diameter of the beam, **Overlap factor (%)** - (1 – stepsize/spot size) x 100, **Overlap factor (k – multiplicative factor)** – determined from the plot shown in the main text **Fig.7(b)**, **Reconstruction pixel size** – Determined from the sample detector distance and pixel size of the camera, **AP** – Alternate projection, **DM** – Difference map, Number following the algorithm represents the number of iteration/cycles (http://ftp.esrf.fr/pub/scisoft/PyNX/doc/scripts/index.html). @The pixel size for stacks varies by +/-0.2 nm depending on the photon energy.

SI-2 Diffraction signal before and after background subtraction

In these measurements which intentionally used low beam intensity, the electronic background is a relatively large fraction of the camera signal. The pattern and magnitude of the background are quite stable. The average of 50 background ('dark') images is recorded regularly. Prior to reconstruction, this background image is subtracted from each of the diffraction images (DI) that constitute a ptychographic image. **Fig. S1** is an example. **Fig. S1a** is a singe DI (on $\log_{10}$ scale) from a ptychographic data set measured when a 1.0 μm beam of 192 eV X-rays hits a BN nanobamboo. The patterning in the annulus (the propagation of the zone plate) is a distorted full field signal. **Fig. S1b** is the background signal (no X-rays) on the same log intensity scale as **S1a**. **Fig S1c** is the difference of the DI (**S1a**) and the background (**S1b**) (log scale). **Figure S1d** compares the histograms of Figs. **S1a, b,c** - see caption for explanation of color coding.

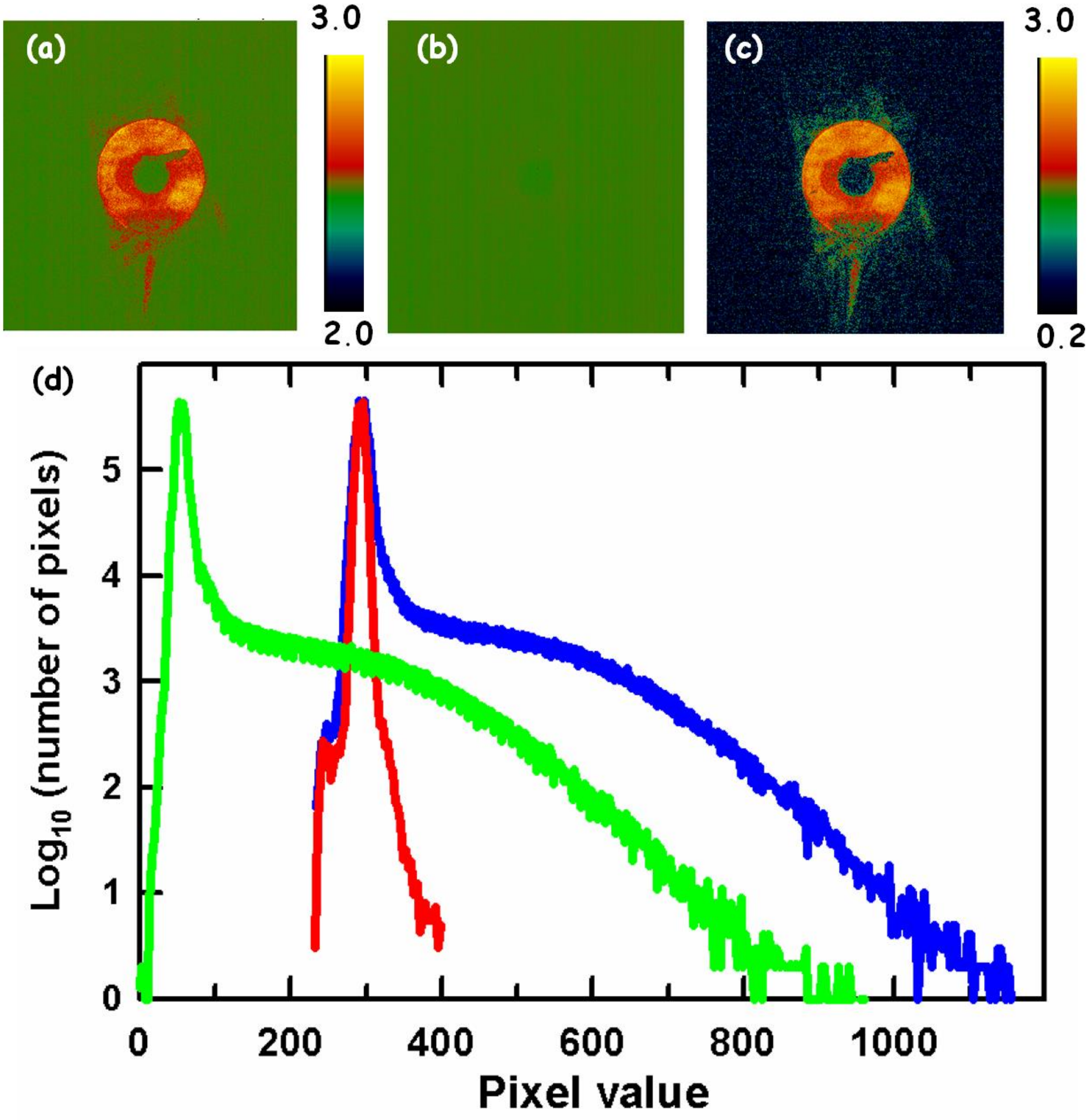

FIG. S1. Example of diffraction images (DI) and processing. (a) DI (on $\log_{10}$ scale) from a BN bamboo measured with a 1.0 μm beam of 192 eV X-rays, 200 ms acquisition. **(b)** is the background signal (no X-rays) on the same scale as **(a)**. **(c)** is the difference of the DI (**S1a**) and the background **(S1b)**. **(d)** displays the histograms of **(a)** in blue, **(b)** in red and **(c)** in green. A threshold cut-off of 20 was applied.

SI-3 Ptychography and spectro-ptychography of carbon nanotubes

Figure S2(a) shows a STXM image of the CNT#1 sample measured at 350 eV using a 25 nm zone plate. **Figure S2(b)** and **S2(c)** show amplitude and phase ptychography images reconstructed from a set of diffraction images (DI) recorded with a 1 µm spot size at 285.2 eV using linear horizontal (LH) polarized light. The dark square in the upper right corner of the ptychography images is carbon build-up from an earlier measurement using a focused spot. The contrast in the phase image is enhanced compared to the amplitude image. One can distinguish between overlapping nanorods due to the strong contrast at the edges of the CNT. **Figure S2(d, e)** show polarization dependent ptychography images measured at 285.2 eV with LV and LH polarization respectively, and normalized to the optical density units. **Figure S2(f)** is the XLD map, obtained as the difference of the ptychography images shown in **Fig. S2(d)** and **Fig. S2(e).** From the XLD map, one can find the CNT with horizontal orientation have white contrast while the CNT with vertical orientation have dark contrast. The XLD contrast of a CNT is strongest at the C 1s → π* transition at 285.2 eV, which has highest intensity when the X-ray polarization is perpendicular to the long axis of the CNT. There is also considerable XLD contrast at the C 1s → σ* transition at 293 eV, which has highest intensity when the X-ray polarization is parallel to the long axis of the CNT. [S1, S2]

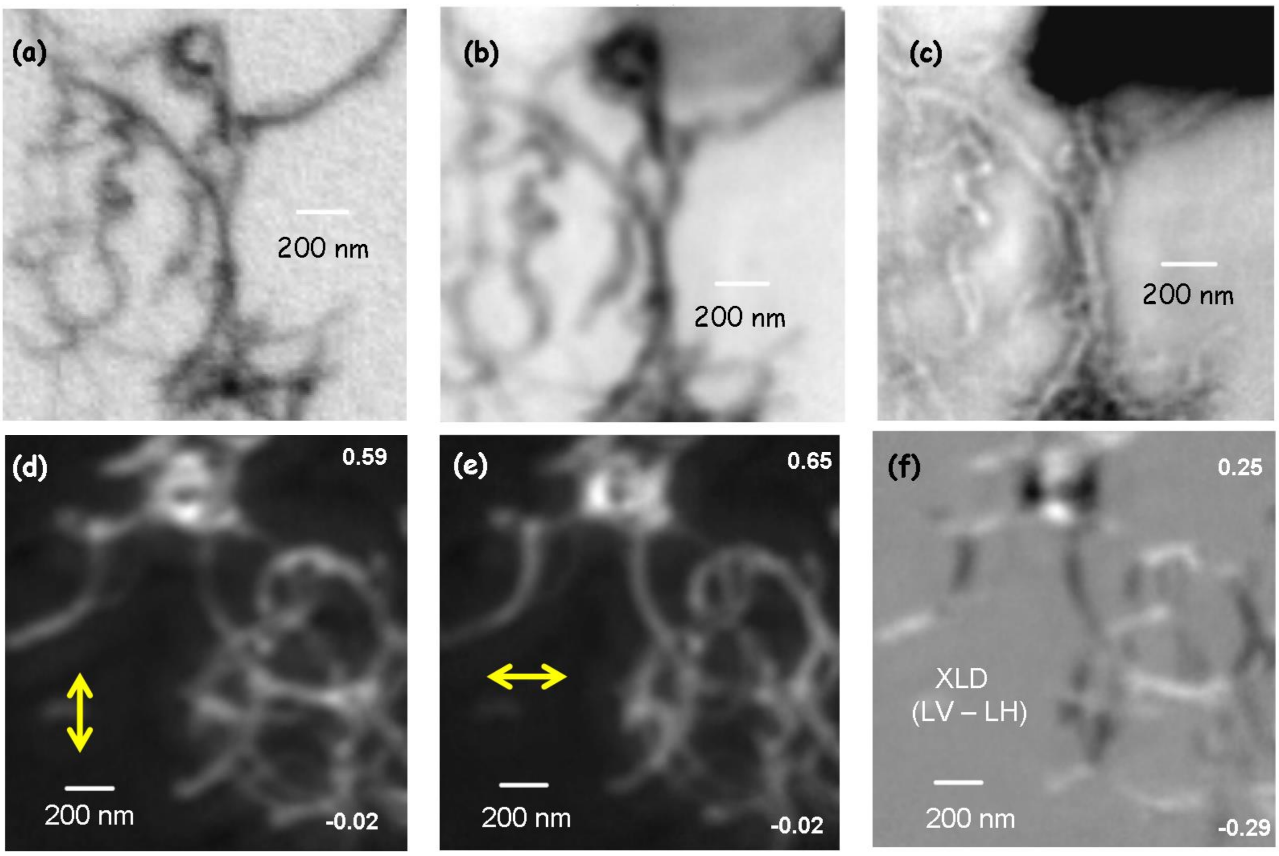

FIG. S2. STXM and ptychography imaging and X-ray linear dichroism of CNT#2. (**a**) STXM image at 350 eV recorded using a 25 nm outer zone, zone plate. (**b**) Ptychography amplitude image and **(c)** phase image of the same region at 285.2 eV recorded using a 50 nm outer zone, zone plate. **(d, e)** Ptychography amplitude measured using LV and LH polarization at 285.2 eV respectively. **(f)** XLD image taken by subtracting **(e)** from **(d).**

For spectro-ptychography we carried out ptychography characterization with photon energies from 280 eV to 304 eV with LV and LH polarization. Then each ptychography amplitude image is reconstructed and normalized to the background. After correcting all the images for the drift with respect to the first image, the pixel intensities highlighted in blue (horizontal tubes) and red (vertical tubes) in **Fig. S3(b)** are plotted as a function of energy from the image sequence to obtain the spectra shown in **Fig.S3(a)**. The blue line indicate absorption when X-ray polarization is parallel to the orientation of the nanotubes and red line (regions) indicate the absorption when X-ray polarization is perpendicular to the orientation of the nanotubes.

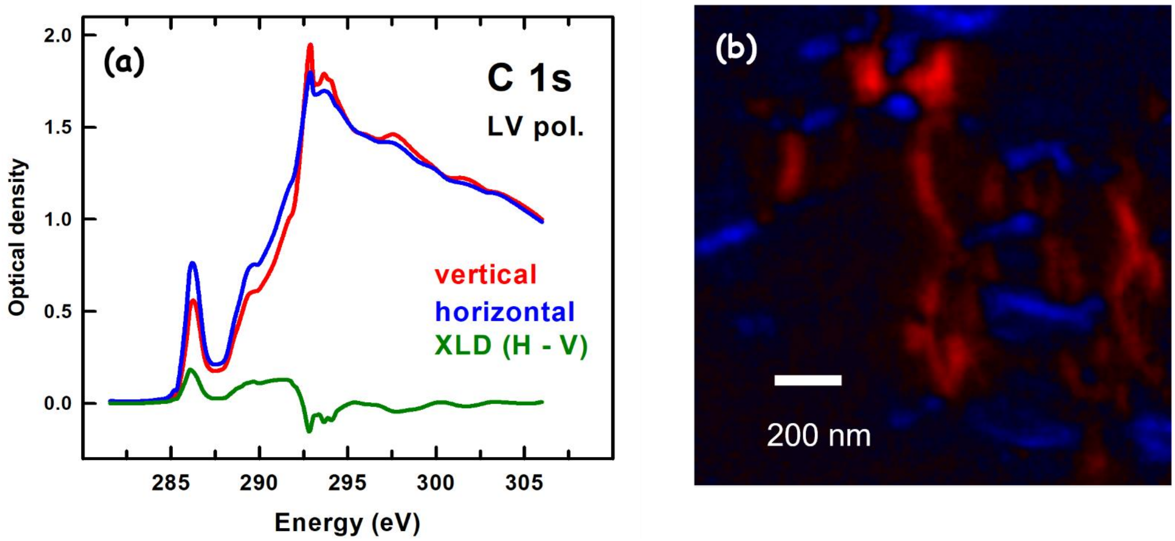

Figure S3: **Spectro-ptychography characterization of C 1s spectra and X-ray linear dichroism (XLD) of AD-CNT #2. (a**) Optical density C 1s spectra measured with LV polarization of vertical (red) and horizontal (blue) oriented CNT derived from amplitude images from ptychographic reconstruction. The green curve is the spectro-XLD signal. **(b**) Color coded composite of the XLD signals where the blue signal is the component map from fit of the stack to the blue spectrum in (**S3a**) and the red signal is obtained from fit of the stack to the red spectrum in (**S3a**).

SI-4 Ptychographic absorption and phase imaging of BN nanobamboo across the B 1s →π*transition

Figure S4 presents absorption and phase images of a region with horizontal and vertical BN nanobamboo over the B 1s →π*transition (191.1 to 192.9 eV).

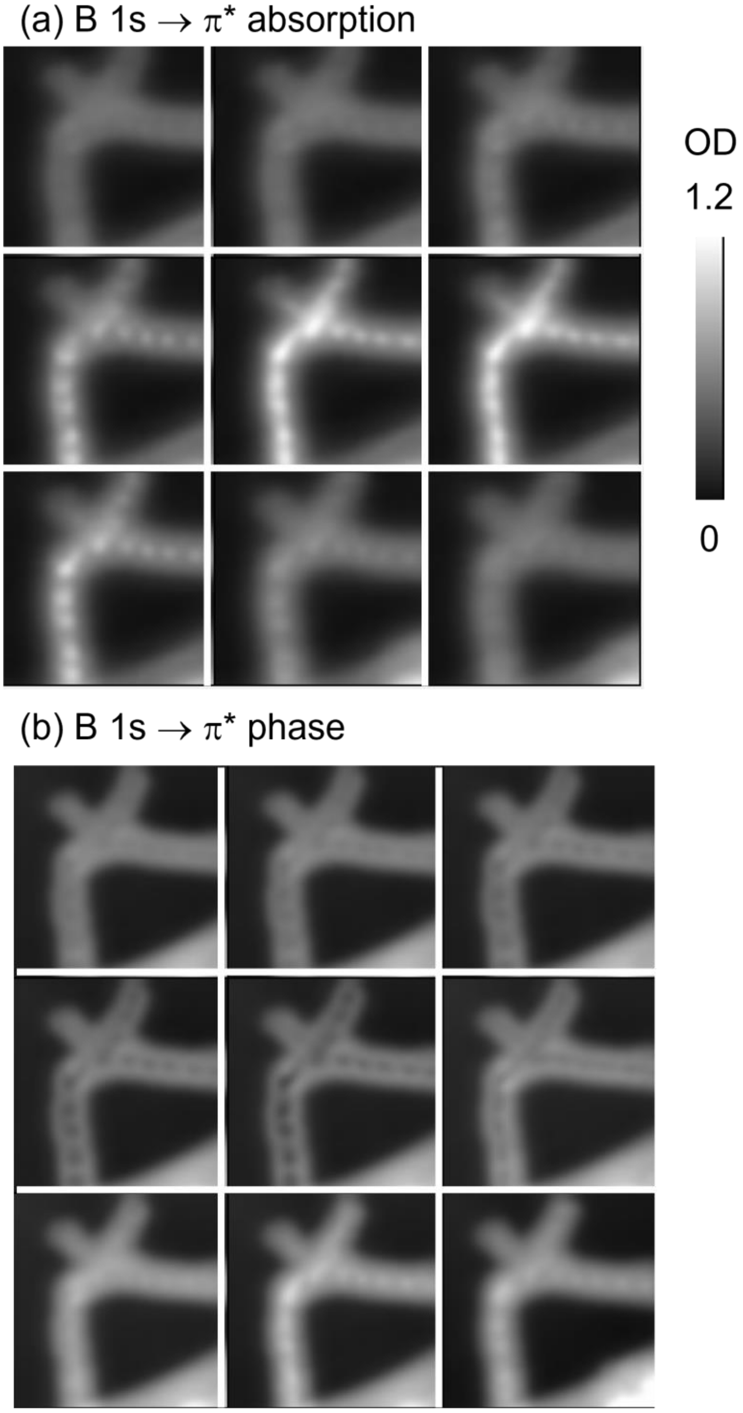

Fig. S4 Absorption and phase imaging of BN nanobamboo across the B 1s →π*transition. (a) Absorption and **(b)** phase images derived from ptychography data measured using LH polarization, 1 μm spot size, 90 % overlap, 100 ms per DI at 9 energies from 191.1 eV to 192.9 in 0.2 eV increments. All images are presented on a common intensity scale, given by the scale bar. Each image is 1050 x 1050 nm.

SI-5 Evaluating spatial resolution

Fig. S5a presents line profile across the edge of a permalloy nanorod from the line indicated in the inset image. The image shown in the inset is the ptychography amplitude image measured at the Fe 2p edge (706 eV) with CL polarization. The y-axis is the amplitude intensity, and the x-axis is position along the length of the line. The abruptness of the decrease in the amplitude across this line (20% – 80 %) is 15 nm, which is an estimate of the resolution of this reconstructed image.

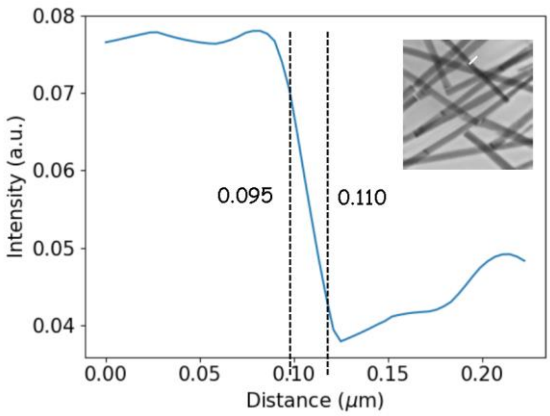

Fig. S5: (a) Line profile of the edge of a permalloy nanorod measured at 706 eV with CL polarization. The white line on the insert image (measured at 706.0 eV with CL polarized light) indicates the location of the line.

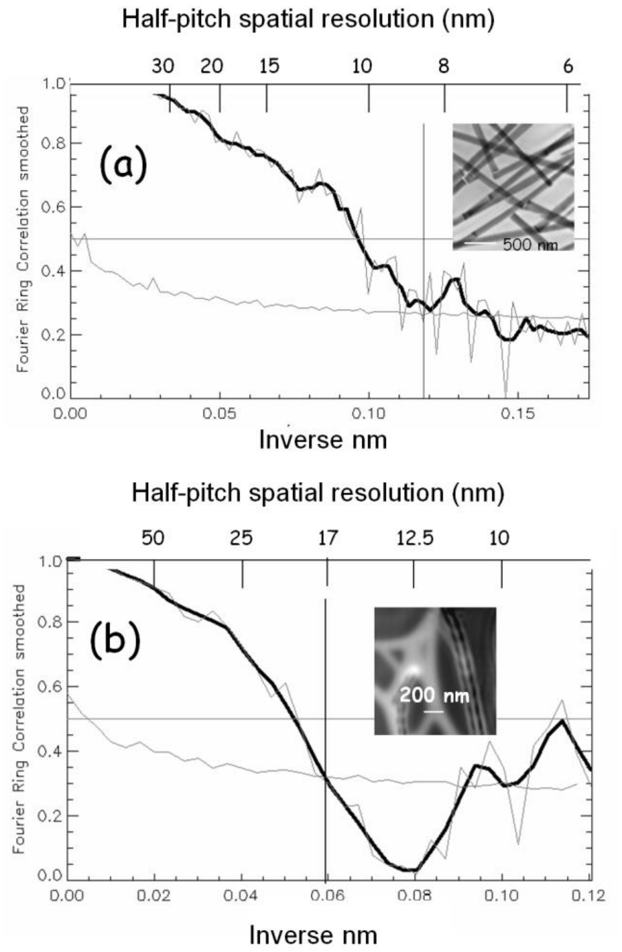

Fig. S6: Fourier ring correlation analysis of the spatial resolution of (a) permalloy sample (amplitude image at 706 eV), and (b) BNB sample (phase image at 192.5 eV. The crossing at the ½-bit line is used to estimate the half-pitch spatial resolution.

Fig. S6 presents the Fourier ring Correlation (FRC) analysis of the spatial resolution for the Fe 2p permalloy sample (**Fig. S6a**, same image as in Fig. **S5**) and the B 1s boron nanobamboo (BNB) sample (**Fig. S6b**, same as Fig. 1e). The crossing of the half-bit curve at 0.12 nm^{-1} indicates a half-pitch spatial resolution of 8.5 nm for the permalloy sample while the crossing of the half-bit curve at 0.060 nm^{-1} indicates a half-pitch spatial resolution of 17 nm in the BNB sample.

SI-6 Comparison of diffraction images from permalloy nanorods and BN nanobamboo

Qualitatively the diffraction images (DI) from the polycrystalline permalloy nanorods are richer and extend to higher q than the DI from the BN nanobamboo (BNB) or CNT samples. However, this is difficult to quantify. In addition, the BNB and CNT data sets were recorded at many different energies and two linear polarizations, whereas the permalloy nanorods were only measured at 706 eV, the maximum of X-ray absorption and XMCD signals. The DI intensities and q-range over which statistically significant signal can be measured from the BNB and CNT strongly depend on the DI acquisition time, as well as the photon energy, polarization, and orientation of the sample relative the E-vector, which is why these samples present rich core level absorption spectroscopy, spectromicroscopy and XLD imaging. **Figure S7** presents a single DI (on $\log_{10}$ scale) from permalloy (**Fig. S7a, S7b**) and from BN (**Fig. S7c, S7d**). The dynamic range of the signal from the permalloy sample is 4 times larger than the dynamic range of the signal from the BNB sample. However, the q-range over which diffraction signal can be seen is similar (see yellow box in **Fig. S7a, Fig. S7b**.

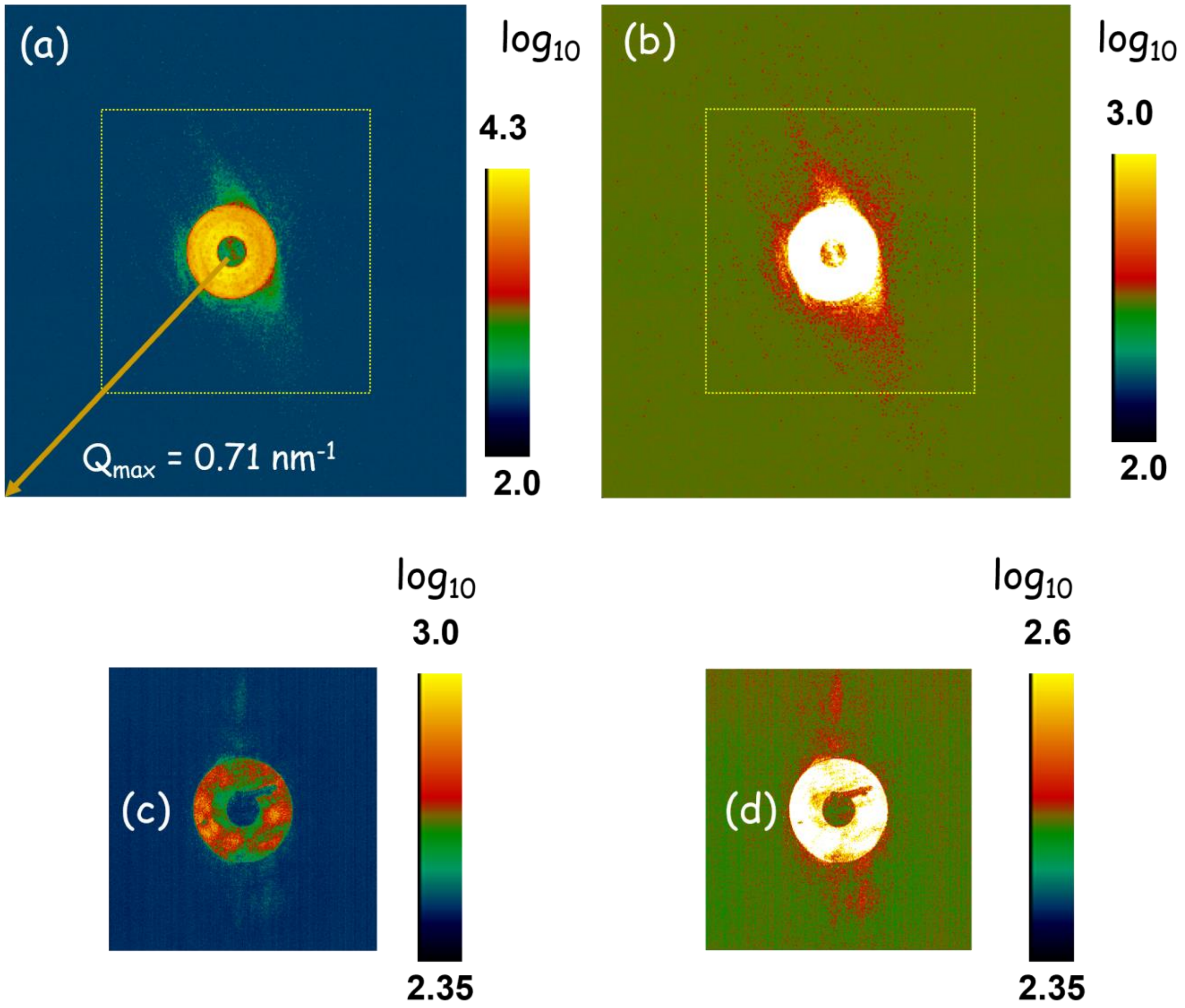

FIG. S7. Scattering signals from a single point. (a) Log_{10} of diffraction Image (DI) measured from the permalloy nanorod at the position marked by “x” in **Fig. 4.a**. (E = 706 eV, q_{max} = 0.71 nm^{-1}). **(b)** Expanded scale of **(a)** to better show the range of diffraction signal. The dotted yellow box in (a), (b) indicates the q-range equivalent to that for the edges of the DI of the BN nanobamboo (c)(d). **(c)** Log_{10} of DI measured from the BNB structure at “x” in **Fig 1d**. (E = 192.0 eV, q_{max} = 0.3 nm^{-1}). **(d)** Expanded scale of (c) to better show the range of diffraction signal.

SI-7 BN nanobamboo measured at N-edge

This image is used to calculate the resolution of the reconstruction of BNB at the N-edge tabulated in Table 1 (in the main text).

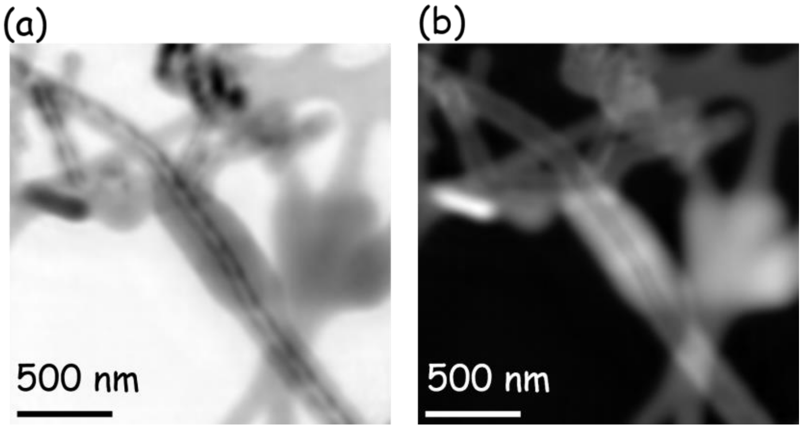

FIG. S8. (a) Amplitude and (b) Phase image of BNB structure measured at 401.8 eV.

References

S1 E. Najafi, D. H. Cruz, M. Obst, A. P. Hitchcock, B. Douhard, J.-J. Pireaux, and A. Felten, “Polarization dependence of the C 1s X-ray absorption spectra of individual multi-walled carbon nanotubes,” Small 4, 2279–2285 (2008).

S2 A. Felten, X. Gillon, M. Gulas, J.-J. Pireaux, X. Ke, G. Van Tendeloo, C. Bittencourt, E. Najafi, and A. P Hitchcock, “Measuring point defect density in individual carbon nanotubes using polarization-dependent X-ray microscopy,” ACS nano 4, 4431–4436 (2010).
